# Mindfulness-based nutritional therapy as a scientific link between nutrition and psychological factors in the inpatient treatment of psychiatric disorders: an intervention study

**DOI:** 10.1192/j.eurpsy.2026.11509

**Published:** 2026-07-31

**Authors:** C. A. Penkov, K. Friedrich, J. Krieger, V. Rößner-Ruff, M. Becksvoort, M. Ziegenbein

**Affiliations:** Wahrendorff Klinikum, Sehnde, Germany

## Abstract

**Introduction:**

Studies and the S3 practice guidelines in psychiatry and psychotherapy show that health-promoting measures are highly relevant in the treatment of mental illness. Current psychiatric care research still shows ambivalent findings regarding the effects of specific diets on psychopathogenic symptoms. In particular, the quality of nutrition and different effects in a psychosomatic context are the focus of studies on different eating habits. Research results outside of biomedical markers are subject to limitations with regard to a psychotherapeutic perspective on nutrition and its influence on mental health. The influence of mindfulness-based interventions in the context of third-wave psychotherapy on the improvement of psychopathogenic symptoms has been comprehensively documented. Mindfulness-related aspects also appear to be significant in the context of nutrition. This intervention study addresses this research gap by examining the influence of mindfulness-centred nutritional therapy on the improvement of mindful eating behaviour, the effects of psychopathogenic symptom severity and structure-promoting nutritional skills, thus taking an alternative, scientific perspective on nutrition and mental health.

**Objectives:**

What influence does mindfulness-based nutritional therapy have on improving psychopathogenic symptoms?

**Methods:**

Following a newly developed, mindfulness-focused nutritional therapy consisting of theoretical and practical units, patients in the intervention group at a specialist psychiatric-psychosomatic hospital completed a four-week therapy unit consisting of several modules. In a measurement taken at three different points in time, a battery of questionnaires was used to examine the influences and modifications of mindfulness and to survey mindfulness-based factors in the context of nutrition, as well as changes in eating patterns towards health-promoting eating behaviour and possible effects on overall psychological stress.

**Results:**

The descriptive and inferential statistical results of the study are presented using regression analyses and multifactorial ANOVA with repeated measurements.

**Conclusions:**

In accordance with the existing research gap, initial conclusions are to be drawn about the possible influence of a mindfulness-based nutritional intervention on improving mindfulness-based nutritional skills and reducing overall symptom severity. Based on the exploration of the possible influence of a multimodal therapy unit, the aim of the study is to investigate the causal effectiveness factors of the application-focused, mindfulness-based nutritional intervention using further influencing variables in a control group setting along nutritional problems of patients with psychopathogenic symptoms, as well as to derive possible implications for the treatment of mental illnesses.

**Disclosure of Interest:**

None Declared

